# Comparison of hemodialysis urea clearance using spent dialysate and Kt/Vurea equations

**DOI:** 10.1111/aor.14848

**Published:** 2024-08-23

**Authors:** Priyanka Khatri, Andrew Davenport

**Affiliations:** ^1^ Fast and Chronic Programmes Alexandra Hospital Singapore Singapore; ^2^ Yong Loo Lin School of Medicine National University of Singapore Singapore Singapore; ^3^ UCL Department of Renal Medicine Royal Free Hospital London UK

**Keywords:** bioimpedance, hemodiafiltration, Kt/V, total body water, urea clearance, Watson equation

## Abstract

**Introduction:**

Dialysis adequacy is traditionally calculated from pre‐ and post‐hemodialysis session serum urea concentrations and expressed as the urea reduction ratio, or Kt/Vurea. However, with increasing hemodiafiltration usage, we wished to determine whether there were any differences between standard Kt/Vurea equations and directly measured spent dialysate urea clearance.

**Methods:**

Urea clearance was measured from collected effluent dialysate and compared with various other methods of Kt/Vurea calculation, including change in total body urea from measuring pre‐ and post‐total body water with bioimpedance and the Watson equation, by standard Kt/V equations, and online clearance measurements using effective ionic dialysance (OLC).

**Results:**

We compared urea clearance in 41 patients, 56.1% male, mean age 69.3 ± 12.6 years with 87.8% treated by hemodiafiltration. Reduction in total body urea was greater when estimating changes in total body urea, compared to measured dialysate losses of 58.4% (48.5–67.6) vs 71.6% (62.1–78), *p* < 0.01. Sessional urea clearance (Kt/Vurea) was greater using the online Solute‐Solver program compared to OLC, median 1.45(1.13–1.75) vs 1.2 (0.93–1.4), and 2nd generation Kt/V equations 1.3 (1.02–1.66), *p* < 0.01, but not different from estimated total body urea clearance 1.36 (1.15–1.73) and dialysate clearance 1.36 (1.07–1.76). The mean bias compared to the Solute‐Solver program was greatest with OLC (−0.25), compared to second‐generation equations (−0.02), estimated total body clearance (−0.02) and measured dialysate clearance (−0.01).

**Conclusion:**

This study demonstrated that the result from equations estimating urea clearance indirectly from pre‐ and postblood samples from hemo‐ and hemodiafiltration treatments was highly correlated with direct measurements of dialysate urea clearance.

## Graphical abstract

We measured urea clearance using standard Kt/Vurea equations with calculation of total body waster using Watson V or bioimpedance analysis and compared the values to Kt/V urea calculated using solute‐solver Lite online program and urea clearance measured from collection of effluent dialysate. Our study demonstrated that the result from equations estimating urea clearance indirectly from pre‐ and post‐blood samples from haemo‐ and haemodiafiltration treatments highly correlated with direct measurements of dialysate urea clearance.

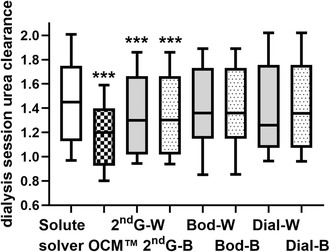

## 1 INTRODUCTION

The amount of dialysis delivered to patients is an important determinant of clinical outcomes for patients with end‐stage kidney disease (ESKD) treated by maintenance dialysis. For many years, dialysis dosing has been based on plasma urea removal and quantified by Kt/Vurea,^1^ adjusting dialyzer urea clearance for differences in body size using the anthropometric Watson equation.^2^ Over time, the equations used to calculate Kt/Vurea have been modified, moving from single pool to dual pool kinetics, and subsequently to allow for different hemodialysis treatment schedules.^3^, ^4^, ^5^

Clinical guideline committees have recommended that the dialysis dose should be measured by a validated method.^6^ In many centers, dialyzer urea clearance has been traditionally measured monthly by blood sampling, although alternative methods determining dialysis dose have now been introduced based on measurements of dialysate conductivity,^5^ or ultraviolet absorbance of the spent dialysate.^7^ As these methods require no blood sampling and can be used to assess dialyzer clearance with every dialysis session, it has been suggested that these advances in dialysis machine technology could potentially replace the traditional pre‐ and postdialysis blood sampling to estimate dialyzer urea clearance.^8^, ^9^

With the emerging evidence for benefits with online hemodiafiltration (HDF) for mortality among ESKD patients, the use of HDF is increasing worldwide.^10^ Kt/Vurea equations were developed for hemodialysis and are extrapolated to HDF treatments in clinical practice. Therefore, it is important to validate these Kt/V equations for HDF treatments. In the modern dialysis era, there have been few if any studies that have actually measured urea removed in the spent dialysate. We therefore wished to compare the urea clearance using the standard Kt/V equations based on pre‐ and post‐treatment blood samples with the actual amount of urea removed in spent dialysate during HDF sessions. It is also well recognized that during hemodialysis (HD), there are changes in the distribution of blood within the body, which can lead to differences in urea concentrations between different body compartments, resulting in the postdialysis urea rebound.^3^, ^6^, ^11^ As such, we aimed to measure urea in spent dialysate and compare this clearance with standard equations of Kt/Vurea and online clearance measurements during both HD and HDF sessions.

## 2 METHODS

In addition to traditional blood‐based methods of measuring Kt/Vurea in adult hemodialysis patients attending for routine outpatient dialysis treatments, we recorded the effective ionic dialysance (EID) of dialysate sodium using online clearance monitoring (OCM™) with the Online Clearance (OLC™; Fresenius Medical Care, Bad Homburg, Germany). Dialysate conductivity was regularly calibrated and delivered dialysate sodium checked by indirect ion electrode (Roche Cobas, analyzer, Basel, Switzerland), having previously been tested by flame photometry and ion electrophoresis.^12^ Spent dialysate was continuously collected during the dialysis session by placing a T‐piece tube in the waste dialysate drain and continuously collected throughout the dialysis session and thoroughly mixed prior to analysis, and the dialysis session timed using the TeamOn™ software.^13^ All dialysis treatments were undertaken using Fresenius 5008 dialysis machines (Fresenius Medical Care, Bad Homburg, Germany), with high flux polysulfone dialyzers (FX series) either in postdilution hemodiafiltration or hemodialysis mode. All patients were anticoagulated with a single bolus dose of tinzaparin (Leo Pharma, Princes Risborough, UK), with a median dose of 2500 (2500–2500) IU.^13^ Dialysate water met ultrapure standards, with a dialysate flow of 500 mL/min and temperature cooled to 35°C–35.5°C. Dialysate biochemistry: sodium 138 mmol/L (136–138); potassium 2.0 mmol/L (1.0–2.0); calcium 1.25 (1.0–1.25) mmol/L; and bicarbonate, acetate, magnesium, and glucose concentrations were set at 32 mmol/L, 3 mmol/L, 0.5 mmol/L, and 5.5 mmol/L, respectively. Serum urea and other biochemistries were measured in a UK‐accredited laboratory (External Quality Assurance (EQA) ISO/IEC 17043) using an automated analyzer (Roche Cobas; Roche Diagnostics, Maidenhead, UK), with the postdialysis samples taken using the slow‐flow method.^6^ Sessional Kt/Vurea was calculated using the online Solute‐Solver software and by standard dual pool methods.^3^, ^6^, ^13^, ^14^ When calculating urea clearance from dialysate losses and changes in total body clearance, the generation of urea component of the 2nd generation Kt/Vurea equation was set to zero.

Patients had standing height measured and weighed pre‐ and postdialysis with calibrated scales. Urea volume of distribution was estimated using the Watson formula^2^ and by multifrequency bioimpedance (InBody S720; InBody Co., Ltd., Seoul, South Korea), both pre‐dialysis and postdialysis after allowing for re‐equilibration in a standardized protocol.^15^, ^16^ Total body urea was estimated by multiplying serum urea and bioimpedance total body water (TBW). We compared effluent urea (estimated by dialysate urea concentrate x dialysis session time x dialysate flow rate) and predialysis total body urea. In addition, we estimated total body urea removal as the difference between respective pre‐ and postdialysis serum urea concentrations and bioimpedance TBW measurements. Patients with amputations, paralyzed limbs, pacemakers, or other implantable cardiac devices were excluded.

### 2.1 Statistical analysis

Results are presented as mean ± standard deviation, or median, and interquartile range, or percentage. Standard statistical analyses included the D'Agostino and Pearson normality test, paired t‐test, Wilcoxon rank sum pair test, analysis of variance (ANOVA) or Kruskal–Wallis for parametric and nonparametric data, with appropriate post hoc correction where appropriate; Pearson correlation was used for univariate analysis; and Bland Altman to determine bias. Statistical analysis was performed using GraphPad Prism (version 10.2; GraphPad Software Inc., San Diego, CA, USA) and Statistical Package for Social Science version 28.0 (IBM Corporation, Armonk, NY, USA), and Analyze‐It (Analyze IT 4.0, Leeds, UK) was used for Bland Altman analysis. Statistical significance was taken at or below the 5% level.

## 3 RESULTS

**FIGURE 1:**
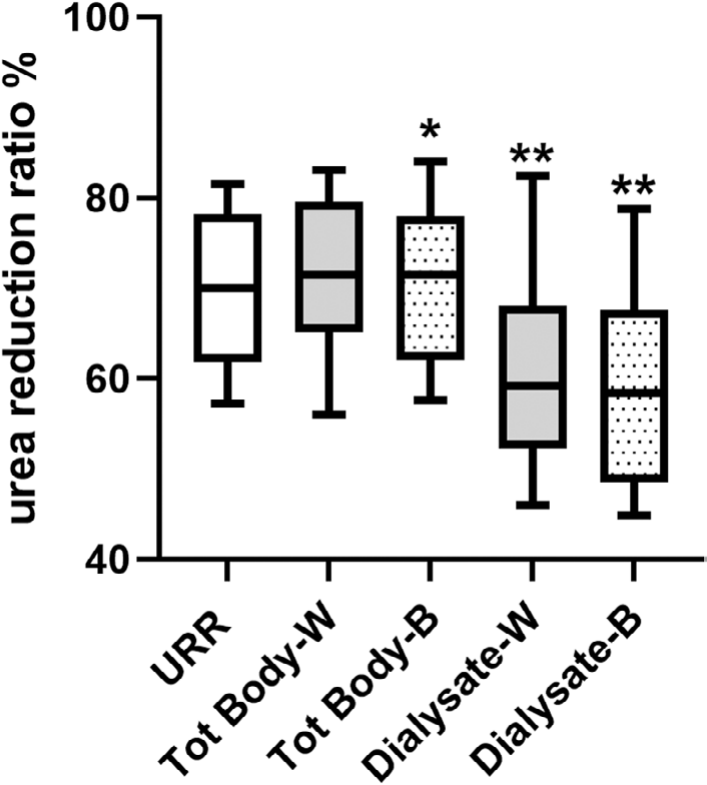
Urea reduction ratio (URR) was calculated from pre‐ and postserum urea concentrations, and from pre‐ and postserum urea and total body water by both the Watson equation (Tot Body‐ W) and bioimpedance (Tot Body‐B), and from dialysate losses and predialysis total body water calculated from Watsons equation (Dialysate‐W) and bioimpedance (Dialysate‐B). Values expressed as median (interquartile range and 90% confidence intervals). *p < 0.05, **p < 0.01 and versus URR. Paired data analysis by Anova (Freidman) with Tukey post hoc adjustment.

**FIGURE 2:**
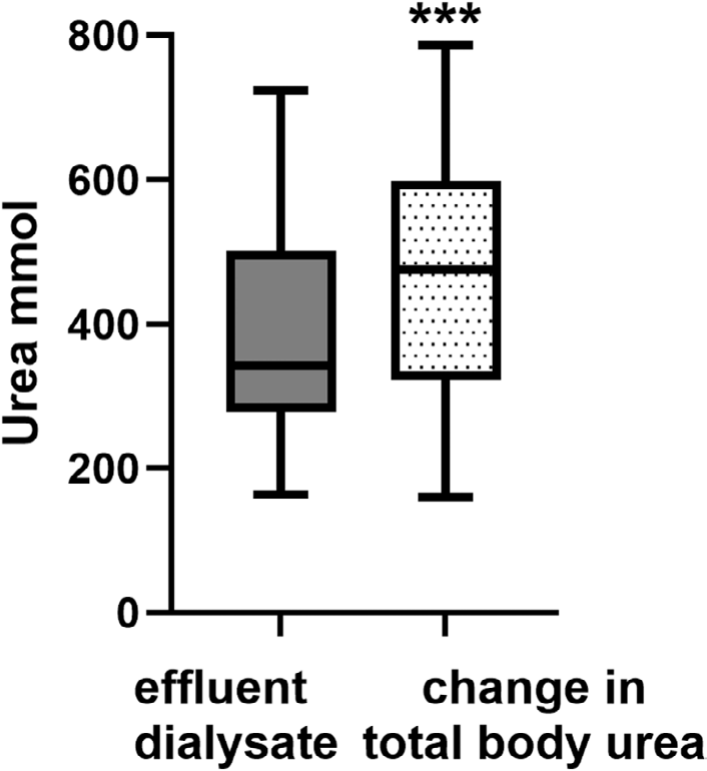
Comparison of the amount of urea measured in the effluent dialysate and that calculated from pre‐ and postdialysis serum urea and pre‐ and postdialysis total body water, respectively. ***p < 0.001 versus. effluent dialysate urea.

**FIGURE 3:**
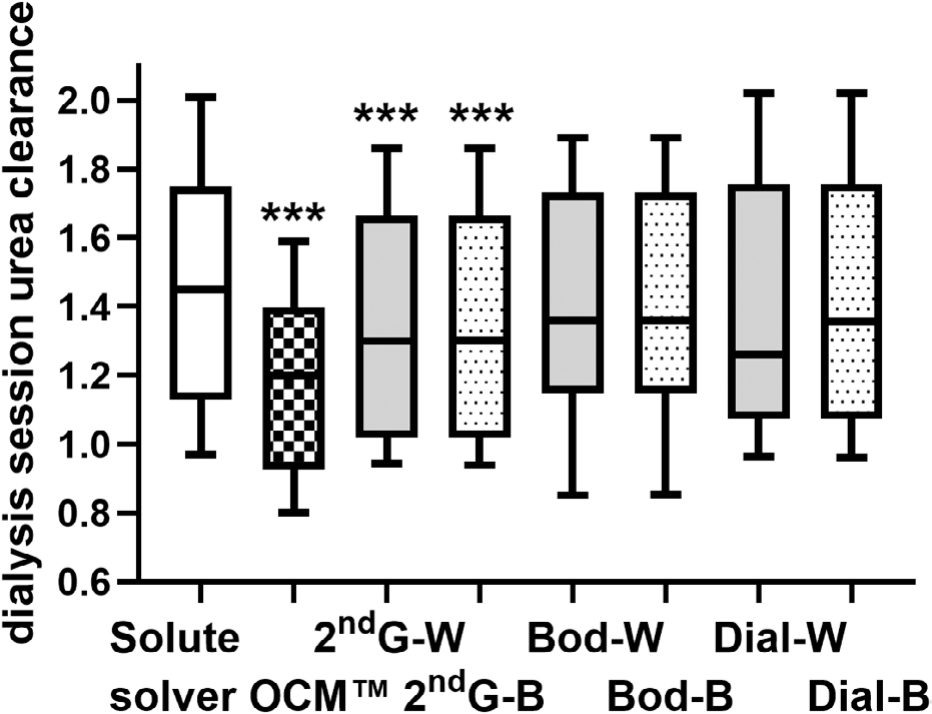
Dialyzer sessional Kt/Vurea calculated from Solute‐Solver program, OCM™ online clearance, Daugirdas 2nd generation equation (2nd G) with both total body water derived from the Watson (W) equation and bioimpedance (B) measurements, and from pre‐ and postserum urea and pre‐ and post‐total body water (Body), and from the effluent dialysate (Dial) ***p < 0.001 versus Solute Solver.

Patients were recruited between April and July 2023, and demographics and dialysis session data are described in Table 1. Urea reduction ratio (URR) was calculated from pre‐ and post hemodialysis (HD) serum urea concentrations, and from pre‐ and post‐TBW by both Watson formula and bioimpedance analysis (BIA), and from dialysate urea losses and predialysis TBW. The Mean TBW was 37 ± 8 liter using the Watson equation and 36 ± 7 liter using BIA. URR was significantly greater than that calculated from dialysate losses and total body losses using bioimpedance (Figure 1). The amount of urea measured in the effluent dialysate was less than that calculated from pre‐ and postdialysis serum urea and pre‐ and postdialysis TBW (Figure 2). Although bioimpedance measured TBW was less than that estimated by the Watson equation, median 33.5 L (29.0–41) versus 35.4 L (30.8–39.5); this was not statistically different, with a mean bias on Bland Altman of 1.0 ± 5.7 L (mean ± 95% limits of agreement). Dialysis session Kt/Vurea was then calculated using Solute‐Solver software, the second generation Daugirdas equation, and the estimated clearance of total body urea and effluent dialysate urea using both Watson and BIA‐derived TBW. Compared to the Solute‐solver sessional Kt/Vurea, the OCM™ online clearance was significantly lower, as was Kt/Vurea calculated using the Daugirdas 2nd generation equation with both TBW derived from the Watson equation and BIA (Figure 3). There were only 5 patients who underwent hemodialysis treatments, and their data were included in the overall analysis. Additional data for this subgroup are presented as Table A1.

**TABLE 1:** Patient demographics, relevant pre‐ and postdialysis blood tests and dialysis session data.

Variables	Results
Number	41
Male/female (%)	56.1/43.9
Age (years)	69.3 ± 12.6
Diabetic (%)	37 (90)
Ethnicity (%)	
White	17 (41)
Asian	12 (29)
African/Afro Caribbean	12 (29)
Predialysis hemoglobin (g/L)	111.2 ± 19.0
Predialysis serum albumin (g/L)	36.2 ± 6.3
Predialysis serum urea (mmol/L)	17.9 ± 6.3
Weight predialysis(kg)	74.2 ± 18.1
Weight postdialysis (kg)	72.6 ± 17.8
Body mass index (kg/m^2^)	26.0 ± 5.8
Blood pump speed (mL/min)	300 (270–300)
Dialyzer surface area (m^2^)	1.8 (1.8–2.2)
Session duration (min)	180 (180–210)
Hemodiafiltration/hemodialysis (%)	87.8/12.2
Convection volume (L)	18.8 (13.4–22.1)
Postdialysis serum urea (mmol/L)	5.6 ± 2.9

*Note*: Values expressed as mean ± standard deviation, median, and interquartile range, or percentage.

**FIGURE A1:**
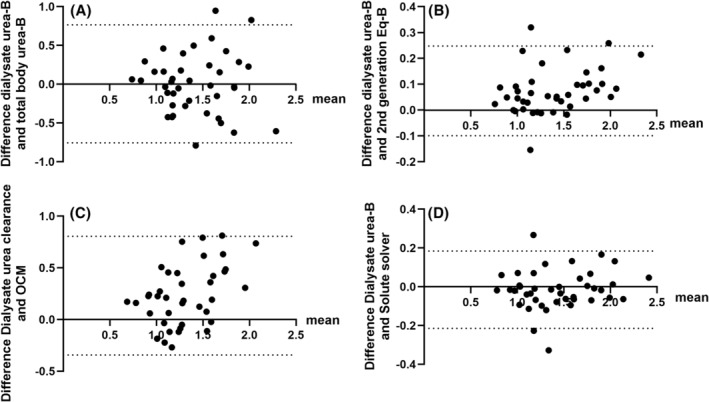
(A) Bland Altman plots of the mean and difference between Kt/Vurea calculated from dialysate urea and total body water (TBW) using bioimpedance (Dialysate urea‐B) and Kt/V values calculated from total body urea and bioimpedance‐derived TBW (Total body urea‐B). (B) Bland Altman plots of the mean and difference between Kt/Vurea calculated from dialysate urea and total body water (TBW) using bioimpedance (Dialysate urea‐B) and Kt/Vurea calculated from 2nd generation Daugirdas equation using TBW derived from bioimpedance (2nd Gen equation‐B). (C) Bland Altman plots of the mean and difference between Kt/Vurea calculated from dialysate urea and total body water (TBW) using bioimpedance (Dialysate urea clearance) and online clearance (OCM). (D) Bland Altman plots of the mean and difference between Kt/Vurea calculated from dialysate urea and total body water (TBW) using bioimpedance (Dialysate urea‐B) and Kt/Vurea calculated from Solute‐Solver program.

On Bland Altman analysis, there was minimal mean bias with most methods of calculating dialysis sessional Kt/Vurea compared to that of the Solute‐Solver software, apart from OCM™ online clearance (Table 2). Kt/V urea calculated from dialysate urea had a good correlation with other methods of calculation. The individual Bland Altman plots depicting difference and mean bias with various methods of Kt/Vurea calculations compared to that from spent dialysate are provided in Appendix (Figure A1).

**TABLE 2:** Bland Altman comparison of sessional urea clearance (Kt/Vurea) derived from effluent dialysate urea clearance and total body water measured by bioimpedance and Solute Solver software program, online clearance monitoring (OCM™), Kt/Vurea calculated from 2nd generation Daugirdas equation using Watson (W) equation or bioimpedance (B) measurement of total body water, and estimated change in total body urea (Total Body) with total body water using Watson or bioimpedance assessments, and univariate correlation (*r* ^2^).

Method	Mean bias	SD bias	95% CL	*r* ^2^	*p*
Solute Solver	−0.01	0.10	−0.21 to 0.02	0.94	<0.001
OCM™	0.23	0.29	−0.34 to 0.81	0.48	<0.001
2nd generation Daugirdas W	0.74	0.09	−0.10 to 0.25	0.95	<0.001
2nd generation Daugirdas B	0.74	0.09	−0.10 to 0.25	0.95	<0.001
Effluent dialysate B	0	0.00	−0.00 to 0.02	0.99	<0.001
Δ total body urea W	0.00	0.39	−0.76 to 0.77	0.29	<0.001
Δ total body urea B	0.01	0.39	−0.76 to 0.77	0.29	<0.001

*Note*: 2nd generation Daugirdas equation.^3^, ^4^

Abbreviations: 95% CL, 95% confidence limits; SD, Standard deviation.

## 4 DISCUSSION

Over time, there have been various modifications of equations designed to estimate dialyzer urea clearance^1^, ^3^, ^5^, ^14^ Accurate assessment of TBW is crucial for the accuracy of these equations. TBW estimation can be done using the Watson equation or BIA. Watson equation, which has been used for the calculation of TBW, was derived in mostly healthy volunteers^17^ and is based on BMI, which does not differentiate between fat and muscle, which have different water contents in dialysis patients. Obesity, malnutrition, low muscle mass, and fluid overload can have significant implications on measurements of TBW.^18^ BIA measurements provide an alternative method of determining TBW, and several devices, including the device used in our study, have been validated in patients of different ethnicities.^19^, ^20^, ^21^ Although the estimates may potentially be less accurate in patients with end‐stage kidney disease, the accuracy can be improved by standardization of protocols to allow for re‐equilibration post dialysis.^22^ Given the potential inaccuracies of the standard methods of blood sampling and use of the Watson equation, it is important to compare the urea clearance estimates using TBW calculations with Watson V and BIA and also to directly compare these estimates of Kt/V with the actual amount of urea removed. There have been very few studies that have reported on measuring urea removed in the effluent dialysate. In part, this is due to the technical difficulties in collecting all the waste dialysate, then effectively mixing the dialysate due to the variation in urea concentrations in different layers that form due to temperature differences, and then cleaning and sterilizing any collecting vessel, as bacterial contamination can introduce ureases, which then affect urea measurements. As such, most previous studies have only reported on batch dialysis systems such as the Genius™ (Fresenius Medical Company, Bad Homberg, Germany).^23^ We developed a method for continuously sampling effluent dialysate,^13^ and measured urea in the effluent dialysate. As expected, the amount of urea removed in the effluent dialysate was less than that calculated from the pre‐ and postdialysis serum urea concentrations and TBW either derived from Watson or BIA. This difference can be explained by differences in urea clearance from different body compartments during the dialysis session, and similarly, the URR calculated from effluent dialysate was lower than that using pre‐ and postdialysis serum urea concentrations.

We chose to compare sessional dialysis urea clearance against the Solute‐Solver software program, as this is the latest iteration of Kt/Vurea equations.^14^ Urea clearance was calculated using the Solute‐Solver LITE single patient calculator (Urea). This online program allows Kt/V calculation using dialysate and blood flows, pre‐ and post‐urea, pre‐ and postweight as well as dialysis time plus KoA of the dialyzer used. The average Kt/Vurea calculated using this method was 1.4 ± 0.4. Urea clearance was greater using the Solute‐Solver program compared to Kt/Vurea calculated using the standard 2nd generation Daugirdas equation, whether V was calculated either by the Watson equation or measured with BIA. Sessional Kt/Vurea using the Solute‐Solver program was also greater compared to EID. While the mean bias calculated by Bland Altman analysis was modest for most methods of Kt/V calculations, it was greater for online clearance, with a suggestion of increasing bias at higher Kt/Vurea clearances. The online EID method measures clearance intermittently during the dialysis session and indirectly by first inducing an increase in the sodium gradient between the serum and dialysate and then measuring the change in dialysate conductivity, with an estimated sodium load of 1.53 ± 7.62 mmol,^24^ followed by alternating pulses with a reduction in the sodium gradient, designed to prevent sodium loading during the dialysis session. The results of previous studies of EID have been varied, with some studies reporting a strong agreement between EID online clearance, and others not.^5^, ^8^, ^25^ Most of the dialysis sessions we studied were in hemodiafiltration mode, and although some studies have not observed a difference between high‐flux hemodialysis and hemodiafiltration,^26^ we used cooled dialysate, and as such this may have affected the estimated online clearance, as the movement of urea and sodium in plasma water may differ at lower temperatures.

Some previous reports have demonstrated differences in Watson and bioimpedance estimates of TBW.^8^ Although we noted a minor difference, this was not statistically significant. There was no difference between any of the dialysis session Kt/Vurea assessments, whether TBW was calculated using the Watson equation or BIA. Other studies have shown that the differences in TBW derived from the Watson equation and bioimpedance measurements are greatest when comparing patients with either very low or high fat mass, as the differences in calculated and measured total body water are due to the differences in water content between adipose tissues (≈10%) and muscle (≈90%).^27^ The median body mass index of our patients was 24.8 kg/m^2^, which could potentially explain the lack of a significant difference between the two measures. Some studies have reported that differences in TBWr affect estimates of clearance, especially with the EID method,^24^ and this was demonstrated by differences between online clearance estimates when comparing Watson and bioimpedance‐derived TBW.^24^ As our dialysis machines used the Watson equation when reporting online clearance, potentially Kt/Vurea clearance may have been slightly higher if we had used BIA‐derived TBW.

As with any study, our results must be taken in context, in that urea clearance during a dialysis session can be affected by many factors, including dialyzer type, effective treatment time, blood and dialysate flows, dialysate composition and temperature, both vascular access type and access recirculation, and cardiac output, and as such, our results may differ from those in other centers with different practices. In addition, our center uses cooled dialysates and lower dialysate sodium concentrations, compared to many others, and whether this may have influenced the EID method remains to be determined.

Apart from studies using batch dialysates, our literature search was unable to identify a previous study directly comparing urea clearance from collections of effluent dialysate and equations based on pre‐ and postdialysis serum urea measurements during hemodialysis and hemodiafiltration sessions. It is therefore reassuring to demonstrate that there is a high degree of reliability, particularly with the latest iteration of the series of Kt/V equations with the Solute‐Solver web‐based software and direct measurements of dialysis session urea clearance, particularly for patients treated by hemodiafiltration.

## AUTHOR CONTRIBUTIONS

PK collected data. AD obtained ethical approval. PK and AD reviewed and approved the submission.

## FUNDING INFORMATION

None.

## CONFLICT OF INTEREST STATEMENT

The authors have no conflict of interest.

## ETHICS APPROVAL STATEMENT

This study was conducted under the approval of the United Kingdom (UK) National Research Ethics Service (approval number 21/NI/0059) and in the spirit of the “Declaration of Helsinki.” All analyses and reporting were conducted in compliance with UK National Health Service (NHS) guidelines for reporting medical studies.

## PATIENT CONSENT STATEMENT

Individual patient consent was obtained in keeping with UK National Research Ethics guidelines in keeping with the Helsinki accord.

## Data Availability

Data are stored on the V drive of the UCL Department of Renal Medicine and may be available at reasonable request under NHS guidelines on data anonymity.

## Appendix

**TABLE A1:** Difference in urea clearance values in patients undergoing hemodialysis versus hemodiafiltration.

Variable	Results (HD)	Results (HDF)
Number	5	36
2nd Gen Daugirdas W (mean ± SD)	1.47 ± 0.47	1.44 ± 0.39
2nd Gen Daugirdas B (mean ± SD)	1.04 ± 0.38	0.89 ± 0.2
OCM (mean ± SD)	1.22 ± 0.33	1.18 ± 0.29
Solute solver (mean ± SD)	1.47 ± 0.48	1.39 ± 0.4
Kt/V dialysate (mean ± SD)	0.80 ± 0.15	0.6 ± 0.14

*Note*: Solute solver: Kt/V urea measurements done using online solute‐Solver LITE single patient calculator (Urea). Kt/V dialysate: Kt/Vurea measurements done by measuring the urea removed in spent dialysate.

Abbreviation: OCM, online clearance monitoring.

Kt/Vurea calculated using 2nd Generation Daugirdas equation with total body water calculated using Watson (W) equation or bioimpedance analysis (B).

## References

[1] Gotch FA . Kt/V is the best dialysis dose parameter. Blood Purif. 2000;18:276–285. 10.1159/000014449 10965068

[2] Watson P , Watson I , Batt R . Total body water volumes for adult males and females estimated from simple anthropometric measurements. Am J Clin Nutr. 1981;33:27–39. 10.1093/ajcn/33.1.27 6986753

[3] Daugirdas JT . Second generation logarithmic estimates of single‐pool variable volume Kt/V: an analysis of error. J Am Soc Nephrol. 1993;4:1205–1213. 10.1681/ASN.V451205 8305648

[4] Butt U , Davenport A , Sridharan S , Farrington K , Vilar E . A practical approach to implementing incremental haemodialysis. J Nephrol. 2024. 10.1007/s40620-024-01939-2 38763995

[5] Raimann JG , Ye X , Kotanko P , Daugirdas JT . Routine Kt/V and normalized protein nitrogen appearance rate determined from conductivity access clearance with infrequent postdialysis serum urea nitrogen measurements. Am J Kidney Dis. 2020;76(1):22–31. 10.1053/j.ajkd.2019.12.007 32220509

[6] Hemodialysis Adequacy 2006 Work Group . Clinical practice guidelines for hemodialysis adequacy, update 2006. Am J Kidney Dis. 2006;48(Suppl. 1):S2–S90. 10.1053/j.ajkd.2006.03.051 16813990

[7] Mohammed A , Davenport A . Comparison of methods to estimate haemodialysis urea clearance. Int J Artif Organs. 2018;41(7):371377. 10.1177/039139881876683 29642725

[8] Lindley EJ , Chamney PW , Wuepper A , Ingles H , Tattersall JE , Will EJ . A comparison of methods for determining urea distribution volume for routine use in on‐line monitoring of haemodialysis adequacy. Nephrol Dial Transplant. 2009;24:211–216. 10.1093/ndt/gfn457 18697799

[9] Davenport A . Moving away from measuring serum urea nitrogen to estimate dialyzer urea clearance: technological panacea or curio? Am J Kidney Dis. 2020;76(1):10–12. 10.1053/j.ajkd.2020.01.017 32362415

[10] Maduell F , Espinosa DE , Broseta JJ . Latest trends in hemodiafiltration. J Clin Med. 2024;13(4):1110. 10.3390/jcm13041110 38398423 PMC10888566

[11] Davenport A . Why is intradialytic hypotension the commonest complication of outpatient dialysis treatments? Kidney Int Rep. 2022;8(3):405–418. 10.1016/j.ekir.2022.10.031 36938081 PMC10014354

[12] Ekbal NJ , Consalus A , Persaud J , Davenport A . Reliability of delivered dialysate sodium concentration. Hemodial Int. 2016;20(Suppl 1):S2–S6. 27669545 10.1111/hdi.12465

[13] Chhabra R , Davenport A . Is increased subjective thirst associated with greater interdialytic weight gains, extracellular fluid and dietary sodium intake? Artif Organs. 2024;48(1):91–97. 10.1111/aor.14657 37902178

[14] Solute‐Solver LITE single patient calculator (Urea). Dialysis adequacy tools and calculators. Accessed 2 Apr 2024. Available from: Ureakinetics.org

[15] Tangvoraphonkchai K , Davenport A . Do bioimpedance measurements of over‐hydration accurately reflect post‐haemodialysis weight changes? Nephron. 2016;133(4):247–252. 10.1159/000447702 27505163

[16] Tangvoraphonkchai K , Davenport A . Pre‐dialysis and post‐dialysis hydration status and N‐terminal pro‐brain natriuretic peptide and survival in haemodialysis patients. Int J Artif Organs. 2016;39(6):282–287. 10.5301/ijao.5000514 27515857

[17] Noori N , Wald R , Sharma A , Goldstein MB . Volume estimates in chronic hemodialysis patients by the Watson equation and bioimpedance spectroscopy and the impact on Kt/Vurea calculation. Can J Kidney Health Dis. 2018;10(5):2054358117750156. 10.1177/2054358117750156 PMC5768265 29348925

[18] Johansen KL , Lee C . Body composition in chronic kidney disease. Curr Opin Nephrol Hypertens. 2015;24(3):268–275. 10.1097/MNH.0000000000000120 25887900 PMC4778545

[19] Blue NMM , Tinsley GM , Hirsch KR , Ryan ED , Ng BK , Ryan SAE . Validity of total body water measured by multi‐frequency bioelectrical impedance devices in a multi‐ethnic sample. Clin Nutr ESPEN. 2023;54:187–193. 36963862 10.1016/j.clnesp.2023.01.026

[20] Blue M , Hirsch K , Brewer G , Cabre H , Gould L , Tinsley G , et al. The validation of contemporary body composition methods in various races and ethnicities. Br J Nutr. 2022;128:2387–2397. 10.1017/S0007114522000368 35109945

[21] Ng BK , Liu YE , Wang W , Kelly TL , Wilson KE , Schoeller DA , et al. Validation of rapid 4‐component body composition assessment with the use of dual‐energy X‐ray absorptiometry and bioelectrical impedance analysis. Am J Clin Nutr. 2018;108(4):708–715. 10.1093/ajcn/nqy158 30099474 PMC7263310

[22] Tangvoraphonkchai K . Davenport a changes in body composition following haemodialysis as assessed by bioimpedance spectroscopy. Eur J Clin Nutr. 2017;71(2):169–172. 10.1038/ejcn.2016.187 27677366

[23] Dhondt A , Eloot S , Wachter DD , Smet RD , Waterloos MA , Glorieux G , et al. Dialysate partitioning in the genius batch hemodialysis system: effect of temperature and solute concentration. Kidney Int. 2005;67(6):2470–2476. 10.1111/j.1523-1755.2005.00356.x 15882294

[24] Kuhlmann U , Goldau R , Samadi N , Graf T , Gross M , Orlandini G , et al. Accuracy and safety of online clearance monitoring based on conductivity variation. Nephrol Dial Transplant. 2001;16:1053–1058. 10.1093/ndt/16.5.1053 11328916

[25] García Testal A , García Maset R , Fornés Ferrer V , Cañada Martínez AJ , Rico Salvador IS , Royo Maicas P , et al. Torregrosa De Juan E. Influential factors on dose by ionic dialysance in daily practice in chronic hemodialysis. Nephrol Ther. 2021;17(2):101–107. 10.1016/j.nephro.2020.11.001 33461895

[26] Maduell F , Puchades MJ , Navarro V , Torregrosa E , Rius A , Sánchez JJ . Monitoring hemodialysis dose with ionic dialisance in on‐line hemodiafiltration. Nefrologia. 2005;25(5):521–526. 16392302

[27] Davenport A . Differences in prescribed Kt/V and delivered haemodialysis dose–why obesity makes a difference to survival for haemodialysis patients when using a ‘one size fits all’ Kt/V target. Nephrol Dial Transplant. 2013;28(Suppl. 4):iv219–iv223. 10.1093/ndt/gft237 23787543

